# Issues in mHealth: Findings From Key Informant Interviews

**DOI:** 10.2196/jmir.1989

**Published:** 2012-10-02

**Authors:** Robyn Whittaker

**Affiliations:** ^1^National Institute for Health InnovationUniversity of AucklandAucklandNew Zealand

**Keywords:** Cellular phone, mobile health, mobile phone

## Abstract

**Background:**

mHealth is enjoying considerable interest and private investment in the United States. A small but growing body of evidence indicates some promise in supporting healthy behavior change and self-management of long-term conditions. The unique benefits mobile phones bring to health initiatives, such as direct access to health information regardless of time or location, may create specific issues for the implementation of such initiatives. Other issues may be shared with general health information technology developments.

**Objective:**

To determine the important issues facing the implementation of mHealth from the perspective of those within the US health system and those working in mHealth in the United States.

**Methods:**

Semistructured interviews were conducted with 27 key informants from across the health and mHealth sectors in the United States. Interviewees were approached directly following an environmental scan of mHealth in the United States or recommendation by those working in mHealth.

**Results:**

The most common issues were privacy and data security, funding, a lack of good examples of the efficacy and cost effectiveness of mHealth in practice, and the need for more high-quality research. The issues are outlined and categorized according to the environment within which they predominantly occur: policy and regulatory environments; the wireless industry; the health system; existing mHealth practice; and research.

**Conclusions:**

Many of these issues could be addressed by making the most of the current US health reform environment, developing a strategic and coordinated approach, and seeking to improve mHealth practice.

## Introduction

mHealth has been most succinctly defined as health-related services delivered via mobile communications devices [1]. While this definition is broad in scope, some feel it may not be particularly helpful to define mHealth as a separate entity because it may lead to logistic or regulatory limits being imposed that could stifle innovative integration into the system. Holt has used the term unplatforms to recognize the fact that people already use a multitude of devices and channels that will continue to evolve and develop [2]. Definitions aside, there appears to be value in delivering health-related information and interventions, and improving access to health services via devices that are personal, intelligent, connected, and always with people [3]. Such delivery channels may enable real-time health advice, prompts, monitoring, feedback, personalized support, and interventions that were not easily provided prior to the almost ubiquitous uptake of mobile phones.

A small but growing body of evidence supports the use of mobile phones in health interventions. These include the use of mobile phones for smoking cessation [4-6], other behavior change programs such as weight management [7-9], and self-management of long-term conditions such as diabetes [10-14]. Text messaging is being used to provide reminders for health care appointments [15,16] and to improve the efficiency of health systems [17]. Text messages are also being used to improve medication adherence [18-22] and to provide health information [23,24]. However, there is relatively little research published on the effectiveness of downloadable applications (apps) or software for mobile phones with computer operating systems (smartphones) [25,26].

In the United States approximately 83% of the population uses mobile phones and 73% of them use text messaging [27]. African Americans and English-speaking Hispanic Americans are more likely than white Americans to use text messaging (76% and 83% vs 70%, respectively), to access the Internet via mobile (56% and 51% vs 39%), to download an app (36% and 36% vs 28%), and to engage in social networking via mobile (39% and 35% vs 25%) [27]. In the past few years, smartphones have become increasingly popular, to the extent that over 35% of US mobile phone users now have a smartphone [27]. In another survey, 17% of cell phone users had used their phones to look for health information online, and 9% had a smartphone app to help them track or monitor their health. However, of those who had any apps on their phone (35% of US adults), only one-quarter said they actually used them [28].

The unique feature of all mobile phones—portable persistent connectivity—brings opportunities for health services but also creates some unique issues facing the implementation of mHealth initiatives. While mHealth is in an early stage of expansion with much hype, private investment (estimated to be more than US $500 million in 2011 [29]), and federal government interest [30], it may be timely to consider a strategic approach to these issues.

This paper summarizes some of the key issues facing the implementation of mHealth in the Unites States based on findings from interviews with people across the health and mHealth sectors. Opportunities to address these issues are then outlined from the author’s perspective.

## Methods

Initial discussions were held by the author, as a 2010/11 Commonwealth Fund Harkness Fellow in Healthcare Policy and Practice, with people in the federal health services and the private mHealth sector. These discussions informed the development of important themes to be covered in the interviews. The themes were the priority and value of mHealth, potential benefits to underserved populations, issues and barriers to mHealth implementation, and issues around mHealth research. These initial discussions, along with an environmental scan, also provided a master list of possible interviewees. This was added to by asking each interviewee for recommendations of other potential key informants. The final master list consisted of approximately 76 individuals. It was decided to initially focus on representatives from several areas: relevant Department of Health and Human Services agencies; integrated health systems; people involved in delivering mHealth services; and academics working in mHealth. Potential interviewees were approached by the researcher directly by email or via email introductions from others. A small number did not respond at all (n = 7), but none of those who responded refused an interview. Some potential interviewees who agreed were never able to complete an interview (n = 6), some encounters became informal discussions instead of interviews (n = 5), and more potential interviewees were never contacted due to time constraints and reaching data saturation (n = 31).

Semistructured interviews were conducted by phone or in person between September 2010 and July 2011. Some themes and questions were prespecified (as above), but other topics were allowed to arise during the interviews. The interviews were recorded and transcribed, and a general thematic analysis was undertaken by the author. This analysis involved identifying all discussions on the prespecified themes and other themes raised by the interviewees. The analysis was followed by coding and categorizing discussions into subthemes. Responses to some prespecified questions were analyzed quantitatively where possible. A report on the findings of the interviews detailed by theme and subthemes was provided to interviewees for comment, and some amendments were made based on their feedback. The author developed the categorization of issues as presented in this paper, and the discussion on opportunities to address these issues.

## Results

A total of 27 key informants participated in semistructured interviews. The interviewees were people interested in mHealth from various federal agencies (n = 10), those from integrated health systems (n = 3), academics working in this area (n = 6), and people working in mHealth companies (n = 4), wireless networks (n = 2), and organizations that sponsor mHealth initiatives or research (n = 2).

### The Key Issues in mHealth Implementation

Key issues arising from the informant interviews have been divided into five areas covering the wider health and wireless environment within which mHealth is implemented: the policy and regulatory environment, the wireless network environment, the health system environment, mHealth in current practice, and mHealth research (see Table 1). Not all of these issues are specific to mHealth, and some are most likely specific to the United States.

**Table 1:** Issues facing the implementation of mHealth raised by key informants.

Area	Issues
Policy and regulatory	Privacy and data security FDA^a ^regulation of mHealth initiatives as medical devices Medical practice across states or countries and with respect to clinical practice roles (eg, prescribing regulations) Bandwidth or spectrum availability
Wireless networks	Compatibility across multiple networks Compatibility across multiple platforms and proprietary systems Cost to the public or end user Coverage in remote areas
Health system	Lack of examples of sustainable business models Lack of reimbursement Lack of understanding of value mHealth may provide Clinical roles accountability and integration into clinical practice Integration into electronic health records and health information systems Competing health information technology priorities and broader opportunity cost
mHealth practice	Lack of knowledge of how to do it well Wrong focus on the technology or on advantaged populations (those who don’t need it) Governance in mHealth Publicly available applications not evaluated and without basis in theory or evidence Stand-alone or siloed initiatives due to existing platforms or proprietary systems
Research	Need for more high-quality research Need to demonstrate efficacy and cost effectiveness Mismatch in pace and flexibility between research and technology development Measurement of reach or access for the underserved

^a ^US Food and Drug Administration.

#### Policy and Regulatory Environment

The issues of privacy and health data security were discussed with all informants. Opinions varied, with some informants considering privacy of personal health information via mobile to be a major issue requiring high-level guidance and widespread discussion before the field can move ahead. A smaller number of informants focused on the potential for straightforward, technologically based security solutions to solve many of the privacy and security issues. The role of the Health Insurance Portability and Accountability Act’s (HIPAA) Privacy, Security and Breach Notification rules was mentioned by most informants (the Act that aims to ensure health plans, health care clearinghouses, and health care providers and their business associates take “reasonable measures” to prevent any uses or disclosures of protected health information that are not permitted or consented to by the individuals). There are some unique aspects of mHealth relating to the Act that have been discussed elsewhere [31]. A further point raised by a small number of informants was the unique position of the wireless networks’ involvement in handling data. Networks have indicated that they are working on technological security solutions.

Another issue raised by the majority of informants was the regulation of mHealth tools and programs as medical devices. Some accepted that regulation is appropriate (and some mHealth developers have proactively sought and received regulatory approval), although some informants expressed concerns, particularly around definitions of what should and what should not require regulation. The US Food and Drug Administration’s draft guidance on this topic was made public after most of these interviews had been completed. This guidance indicates that general health and wellness applications are unlikely to be regulated; nor are applications “not intended for curing, treating, seeking treatment for mitigating, or diagnosing a specific disease, disorder, patient state, or any specific, identifiable health condition” [32]. As the field moves toward more comprehensive and integrated solutions that include mHealth, exactly where the line will be drawn may be less than obvious [33]. Some also expressed a concern that the ongoing iterations and improvements common in this type of agile technology development should not necessitate frequent updates to regulatory approval.

#### Wireless Network Environment

Issues raised in this category mainly came from interviewees involved in implementing mHealth initiatives in a variety of contexts. They commented on the large number of wireless networks in the United States, which can make establishing relationships and interfaces for comprehensive implementation difficult. One example of how this can successfully work was raised by several informants. CTIA-The Wireless Association, a nonprofit member organization that represents the industry, was involved in establishing industry-wide support for the national text4baby service, thereby ensuring that health information text messages for pregnant women and new mothers are available and free of charge regardless of network.

Informants involved in implementing mHealth initiatives discussed the fact that individuals are charged for receiving text messages as an issue. The cost of mHealth services to the public is of particular concern to those working with socioeconomically disadvantaged populations. The cost of mHealth was linked to the wider issue of the use of mHealth initiatives to reach underserved populations, which was uniformly recognized as a potential benefit due to the high levels of mobile phone ownership and use of text messaging in ethnic minority and low socioeconomic populations in the United States [27,34]. However there is, as yet, little published evidence that mHealth has actually improved access to health information or services by those who were not otherwise receiving the service. Some informants also mentioned that a focus on effectively using the ubiquitous aspects of mobile technologies (such as text messaging) for wider reach into populations that need it most contradicts a more popular emphasis on high-tech developments. A small number of informants felt there may be other issues around the use of mobile technologies by disadvantaged populations (for example, around language, culture, or network coverage) that can also affect the ability of mHealth initiatives to reach those in need [35].

Some informants mentioned a barrier to the development of more widely available and more comprehensive solutions arising from proprietary systems and multiple platforms. This is a phenomenon previously seen in other areas of information technology development [36].

#### Health System Environment

The predominant fee-for-service reimbursement structure of the US health care system is seen by almost all informants as less than ideal for the implementation of mHealth. Indeed, many mHealth initiatives aim to reduce in-person reimbursable visits for hospitals, clinics, and providers [6,19,25]. This is obviously a wider issue involving other forms of digital health, telehealth services, and preventive services.

The issue of funding was often linked in informant discussions to a stated lack of understanding of the potential of mHealth to contribute to the health system by funders, managers, and decision makers. This understanding was said to be slow to evolve, and some felt this to be due to a lack of good cost-effectiveness evidence to inform decisions.

Some informants did raise the current lack of demonstrably sustainable mHealth business models in the system. Suggested possible funders of mHealth initiatives were employers, health insurers, individuals, and the federal government. Specific examples discussed were recent private investments in mHealth start-up companies and the national-level public–private partnership behind the text4baby program. However, several interviewees mentioned that more urgent priorities within the health system are monopolizing focus and resources, particularly around the implementation of electronic health records (EHR) systems. Integration issues with EHR systems were also seen as a barrier for those working in the field.

A small number of informants discussed the need for a strategic framework and governance of the implementation of mHealth. This was particularly in the context of issues such as interoperability and standardization, but also with respect to changes in the way health care is delivered. For example, if mHealth becomes a catalyst in moving the locus of control or responsibility for health (and health information) toward patients, then existing governance systems may need to change accordingly. In a similar vein, clinicians’ concerns about mHealth were mentioned by a small number of informants. This was not around adoption of mobile technology per se (doctors are disproportionately high adopters [37]), but more around their roles and accountabilities in this new model of delivery. The examples discussed included responding to direct messages from patients (in various formats) and to real-time, continuous, remote monitoring data, where there are no existing protocols for interpreting this intensity of data. This was expressed as a desire of clinicians to see mHealth implementation being well thought through and any potential adverse consequences addressed in advance.

#### Current mHealth Practice

Several informants raised the concern that many mHealth applications available in practice may not be effective, engaging, usable, or meeting the needs of users. Few applications have been evaluated, and those that have often involve complex interventions where the components or mechanisms have not been examined. Many felt that not a lot is known as yet about what aspects of mHealth work, for whom, and why. Few published health interventions delivered via mobile technologies discuss a theoretical basis or evaluate theoretical components hypothesized to be important in the intervention [38]. It was stated that there is much hype and lots of players all “doing their own thing.” Some informants felt that some mHealth developers may have a bias toward developing programs for people like themselves using the technologies they like, rather than starting with the problem and working with end users to develop the most useful and usable solution. Some pointed to statistics in the media showing that many smartphone applications are downloaded but not used. More recently, reviews have found poor quality in terms of accuracy, usability, consistency with national practice guidelines, and effective practices [26,27,39,40].

#### mHealth Research

When key informants were asked about mHealth research, many rated the state of the evidence as early or weak and identified various areas as being in need of more high-quality research. Most felt that there is sufficient proof that mHealth is worth pursuing, although more solid evidence is required in terms of cost effectiveness and in determining what works. Some felt quite strongly that there should be no need to wait for randomized controlled trials to provide evidence on the effectiveness of every aspect of mHealth before any progress can be made. Two examples given were (1) where there is no access or very limited access to a health service and we can extend access to that service via mHealth with very little possibility of harm, and (2) where mobile delivery can be seen as the natural extension of what we already do, such as in health communication using the current preferred methods of communicating with the population.

Many talked of the obvious mismatch in pace and flexibility between traditional health research methods and rapid technological development. This mismatch was compared with the way research and development are often undertaken concurrently and iteratively in the academic engineering and computer science fields and in commercial development. Since the time of the interviews, the issue of alternative methodologies, appropriate comparators, and standard measures in mHealth research has been under review by research and funding agencies in the United States [41,42].

### Opportunities to Address the Issues

Some interviewees did discuss their thoughts on how these issues should be addressed, although these discussions were not included in every interview. During the analysis of the discussions, the author developed a list of potential opportunities to address the issues in each category (Table 2). These opportunities are summarized in three main areas below.

**Table 2:** Opportunities to address issues in mHealth implementation.

Area	Opportunities
Policy and regulatory	Federal-level guidance Coordination of wireless industry and health sector
Wireless networks	Shared standards for interoperability Consideration of open architecture or standardized interfaces Industry coordination and collaboration for mHealth
Health system	Use of opportunities of current health reform investment (eg, CMMI^a ^demonstration projects; competition in electronic health records industry; beacon community projects and evaluation)
mHealth practice	Collaboration with end users to develop solutions to their problems Foundation of theory and evidence of what works Collaborations and shared learning for iteration and improvement, integration of public data, and integration into health systems Consideration of open source & other methods to reduce barriers to more comprehensive integrated initiatives
Research	Consideration of alternative research methods to increase pace and retain rigor, including careful consideration of comparators Inclusion of measures of increased access Publication of formative research & evaluations of existing interventions

^a ^Center for Medicare & Medicaid Innovation.

#### Health Reform Opportunities

The US health reform environment includes a focus on investment in health information technology and consideration of alternative models of payment and health care provision. This environment creates opportunities to demonstrate the value of mHealth and to ensure that future capabilities are integrated into health information technology systems. For example, the Center for Medicare & Medicaid Innovation “has the resources and flexibility to rapidly test innovative care and payment models and encourage widespread adoption of practices that deliver better health care at lower cost” [43]. Their process is to solicit ideas for new payment and service delivery models, select the most promising models, test and evaluate them, and then spread the successful models. mHealth seems an appropriate means to support their stated priority areas, including bundled payments for patient care, seamless coordinated care, and community and population health models to keep people healthy.

A total of 17 beacon communities, identified as those that have already adopted EHR systems and health information exchange, have been awarded extra funding via the Health Information Technology for Economic and Clinical Health (HITECH) Act to demonstrate the transformative ability of health information technology with respect to quality, cost efficiency, and population health [44]. These communities already include engaged and connected health services, clinicians, and community health workers who have agreed to implement combinations of innovative interventions, to measure their performance, and to share their learnings [45]. These enthusiasts could be encouraged to develop and evaluate mHealth initiatives that make sense for their local health improvement goals, thus helping to demonstrate the efficacy of such initiatives in practice.

Hundreds of EHR system vendors have had an even greater number of products certified by the Office of the National Coordinator for Health Information Technology [46]. This competitive environment could be used to push for the integration of mobile functionality as a point of difference between systems, ensuring that future capability is built into systems being adopted now, instead of as later add-ons.

#### Federal-Level Guidance

A degree of national-level guidance may be warranted, particularly in terms of coordinating a strategic approach to some of the wider issues. This guidance could include coordinating efforts across the wireless and mHealth-related industries and the health sector around topics such as developing common standards and interfaces to allow interoperability; considering privacy and data security solutions; and discussing the movement toward an open architecture and the ability to innovate and integrate across platforms and across networks. Estrin and Sim have stated that mHealth should learn from the Internet’s development and from the previous siloed approaches to health information systems, and should work together toward an ecosystem for innovation [36]. mHealth need not be confined by geographic borders and, if the ecosystem is global, there may need to be international agreement on approaches to these issues. Elsewhere, the adoption of international standards has been facilitated by organizations such as the International Telecommunication Union.

#### Improving mHealth Practice and Research

The need to improve the development of mHealth initiatives came through quite strongly in these interviews. We have the opportunity early in the growth of this field to consider a structured approach to development that is guided by relevant theory and current evidence, and that may lead us to a logical framework or model of how, why, and what works in mHealth. Riley et al suggest that current health behavior theories may not be adequate for interactive and adaptive mobile technology-enabled interventions [38]. Perhaps more research is required on new theories that may advance our understanding of how mHealth initiatives can be effective.

The development process, as in many other fields, needs to start with the problem and work with the end users (clinicians, health service providers, patients, and the general public) to develop the most appropriate and useful solution to that problem. Real-world implementation of initiatives should be considered from the outset, so that practical issues such as intellectual property, scaling up, and integration into practice are addressed. With a more open philosophy, it may be possible to iterate, adapt, and improve on what others have done. This is not always easy, but the end goal should be integration with other health information technology systems, particularly with access to personal health information and publicly available information, in order to develop seamless services that are centered on individuals rather than providers or locations.

Evaluations of effectiveness and usability are required and should be made publicly available. Where evaluation is planned during the development stage, data collection can be built in as an integral part of the program. The ideal of randomized controlled trials will still be necessary in some contexts. In these cases, careful consideration should be given to the appropriate comparator to ensure the right question is being answered. For example, what is usual care for this target audience? Can we measure an improvement in access as an outcome? Other research methods will be more appropriate in other circumstances, such as adaptive trials to allow the intervention to develop and improve as part of the research; observational trials and qualitative research methods to detect unintended consequences and changes to workflow; and qualitative studies to test acceptability. Evaluating effectiveness and usability is also possible while implementing a system, for example, with novel designs such as the stepped wedge cluster randomized trial, and particularly where there is little likelihood of harm.

The other great opportunity for mHealth in practice and research is data collection in environmental and population health surveillance, as early warning systems of public health issues and as emergency information systems in natural disasters or pandemics. Many such systems are being developed and will surely advance the body of knowledge around using mHealth for public health.

## Discussion

This paper outlines key issues in the implementation of mHealth in the United States as raised by an environmental scan and key informants from the health and mHealth sectors. The issues have been categorized according to policy environment, wireless environment, health system, mHealth practice, and research, although many issues could be seen to cut across several categories. This categorization allowed a matching of issues with current opportunities to address them, which were then aggregated into three main action areas: using the opportunities provided by the current health care reform investment and processes; establishing some degree of federal-level guidance and coordination across the industry; and improving mHealth practice with good intervention development principles and sound research methodology.

Despite the large number of issues raised, most informants were optimistic about our ability to address these issues in the near future. These optimists included those who see the transformative potential of mHealth to change the way we deliver health-related services, as well as those who see the use of mobile communications devices as just the natural evolution of what we currently do in health communication and health care.

There are limitations to this study. Although no further new themes were being added, the list of possible issues presented here still may not be exhaustive due to the selection of interviewees. The qualitative analysis of the transcribed interviews was undertaken by the author only. Reflexivity may be an issue, as the author is a researcher in the field of mHealth and therefore not a completely independent observer. However, prior to this period the author had not worked in the United States or with any of the interviewees. It is possible that the initial environmental scan and the author’s own expertise in this area may even have brought some degree of validity filtering to the discussions and the analysis. The categorization of issues and the identified opportunities for addressing them are the opinions of the author only.

### Conclusions

To many people, the use of mobile phones in health is obvious—if their hairdresser is sending them text message reminders, why isn’t their doctor? To date, we have failed to develop mHealth initiatives that are so easy to use and so obviously useful that large numbers of people want to regularly use them over time. As one informant noted, once that is achieved many of the other issues will be resolved.

The overall opportunity of mHealth will come from accessing large, widespread populations directly with individually tailored programs. Even where these programs have only a small effect, with such wide reach there is the opportunity for population-level changes—moving the bell-shaped curve of health a small amount with huge public health impact [47].

## Abbreviations

EHR: electronic health records
HIPAA: Health Insurance Portability and Accountability Act
HITECH: Health Information Technology for Economic and Clinical Health
